# An Adolescent with Transient Hyperthyroxinemia after Blunt Trauma to Head and Neck

**DOI:** 10.1155/2021/6628035

**Published:** 2021-04-08

**Authors:** Michelle Romijn, Leo M. G. Geeraedts, Jonathan I. M. L. Verbeke, Martijn J. J. Finken

**Affiliations:** ^1^Department of Pediatric Endocrinology, Emma Children's Hospital, Amsterdam UMC, Amsterdam, Netherlands; ^2^Department of Surgery, Section Trauma Surgery Amsterdam UMC, Location VUmc, Amsterdam, Netherlands; ^3^Department of Radiology and Nuclear Medicine, Amsterdam UMC, Location VUmc, Amsterdam, Netherlands

## Abstract

**Background:**

Thyroid storm is a well-known complication of surgical procedures in the lower neck, but is rare after a blunt neck trauma. The cases described previously have mainly focussed on adults with pre-existent thyroid disease. In this case report, we describe the disease course of a previously healthy adolescent who had asymptomatic hyperthyroxinemia after a blunt trauma of the jaw and neck. *Case Presentation*. A 17-year-old girl presented at our emergency department after she fell on her head while roller blading. On physical examination, among other injuries, she had a swelling in the lower neck, which appeared to involve the thyroid gland. Subsequent laboratory analysis was indicative of primary hyperthyroxinemia, with a free T4 of 59 pmol/L (reference range: 12–22) and a TSH of 0.46 mU/L (reference range: 0.5–4.3), but the patient had no symptoms fitting with this. Four weeks after the initial presentation, the patient reported only complaints regarding tenderness in the jaw and neck region. She was no longer hyperthyroidic on biochemical evaluation (with a free T4 level of 15.6 pmol/L and a TSH level of 0.33 mU/L), and antibodies against thyroid peroxidase or TSH receptor were not present.

**Conclusions:**

This case might indicate that hyperthyroxinemia following a neck trauma may go unnoticed if hyperthyroid symptoms are mild or absent and thyroid function tests are not performed.

## 1. Introduction

Thyroid storm is a well-known complication of surgical manipulation of the thyroid gland, for example, during parathyroidectomy or laryngectomy [1–5]. However, thyroid storm induced by thyroid injury after blunt trauma to the neck is a rare finding [6, 7]. This clinical entity has been described following attempted suicide by hanging [8, 9], a severe neck trauma [6, 10], and thyroid ultrasonography [11, 12]. A great majority of patients described so far were adults, of whom the majority experienced severe hyperthyroid symptomatology and were known with pre-existent thyroid disease [6, 7, 13]. Thyroid storm is a potentially life-threatening complication of thyroid injury that starts abruptly and is characterized by four main symptom clusters, including fever, supraventricular arrhythmias or tachycardia, and gastrointestinal system and central nervous system symptomatology. Early recognition and prompt treatment seem necessary [6]. In this case report, we describe the disease course of a previously healthy adolescent who had asymptomatic hyperthyroxinemia after a blunt trauma to the head and neck.

## 2. Case Presentation

A previously healthy, 17-year-old girl presented at the emergency department after she fell on her head while roller blading. She complained about pain in her jaw and neck. On physical examination, the patient was an alert girl with stable vital parameters, including a heart rate of 70 bpm, a blood pressure of 125/85 mmHg, a peripheral oxygen saturation of 99% on room air, and a temperature of 37.6°C. Injuries noted were a fractured mandible and a painless, mobile swelling in the lower neck at the level of the thyroid region. There was no audible stridor or other signs of upper airway obstruction.

**Figure 1:**
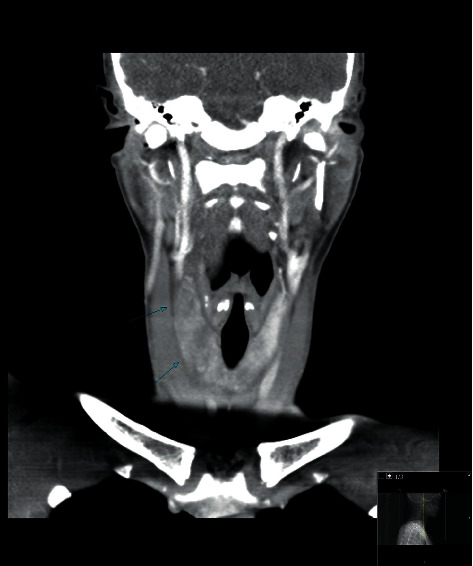
Contrast-enhanced CT image of head and neck, demonstrating thyroid enlargement and asymmetry. After the administration of contrast, the right lobe contained more hypodense areas without enhancement compared to the left lobe, as indicated by the arrows. There was no extravasation of the contrast medium. These findings are suspicious of contusion or laceration, though the possibility of autoimmune thyroiditis could not be excluded.

**Figure 2:**
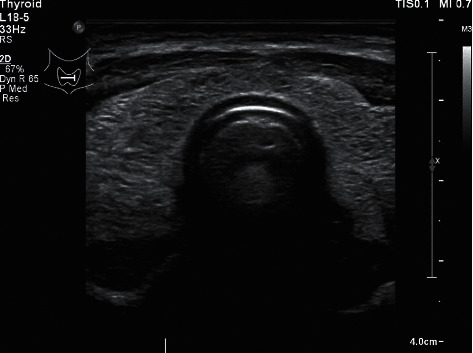
Thyroid ultrasonography of the patient demonstrating thyroid enlargement and inhomogeneity. The size of the left lobe was 1.5 × 1.6 × 2.8 cm, and the size of the right lobe was 1.7 × 1.5 × 3.5 cm. Anterior from both thyroid lobes was fluid accumulation.

Computed tomography (CT) scanning of the head and neck was performed, showing an enlarged and inhomogeneous thyroid gland and a fractured mandible (see Figure 1). Subsequently, contrast-enhanced CT scanning and ultrasonography of the neck showed enlargement and asymmetry of the thyroid gland, with more hypodense areas in the right lobe, suspicious of contusion or laceration, though the possibility of autoimmune thyroiditis could not be excluded (see Figure 2).

After the imaging results, thyroid function tests were performed, demonstrating a free T4 level of 59.0 pmol/L (reference range: 12–22) and a TSH level of 0.46 mU/L (reference range: 0.5–4.3). The patient did not experience symptoms of hyperthyroidism, in particular no jittery, no palpitations, no excessive sweating, and no previous weight loss. She had no history of a neck swelling. The family history was unremarkable, with no family members reporting a history of thyroid or autoimmune disease. The day after admission, the laboratory tests were repeated, showing a decline in the free T4 level (see Table 1). The patient still did not experience symptoms of hyperthyroidism, and 24 hours later, she was discharged.

At an outpatient visit 4 weeks after presentation, the patient reported to be nearly recovered, except for tenderness in her jaw and neck region. On physical examination, her vital signs were stable and her thyroid gland was no longer enlarged. Thyroid function tests showed a free T4 level of 15.6 pmol/L and TSH level that was still slightly suppressed at 0.33 mU/L and absence of antibodies against thyroid peroxidase or TSH receptor (see Table 1). The transient hyperthyroxinemia could therefore most likely be attributed to thyroid injury.

## 3. Discussion

We have presented a patient with transient, asymptomatic hyperthyroxinemia after a blunt trauma of the head and neck. The pathomechanism of trauma-induced hyperthyroxinemia is thought to involve rupture of acini and liberation of thyroid hormones in the bloodstream, which may result in a potentially life-threatening condition called thyroid storm [6]. Thyroid storm is a rare finding among trauma patients that has previously been described only in those with a severe trauma to the neck, such as after hanging and strangulation [8, 9]. There are only a few reports describing thyroid injury and thyroid storm after a blunt neck trauma [6, 7, 10]. Most of the cases described before were adult females, with a median age of 44 (range: 8–89) years, and approximately half of them were known with pre-existing thyroid disease [7]. Newer theories suggest that thyroid storm represents a form of allostatic failure in situations, where severe illness would normally result in euthyroid sick syndrome, but where the patient is unable to downregulate T3 concentrations due to thyrotoxicosis [14]. However, in our patient, T3 concentrations were not measured.

Another complication of thyroid injury is a rapidly expanding hematoma that may cause airway compression requiring hemithyroidectomy. It has been described that nearly half of the patients with thyroid injury required hemithyroidectomy due to a rapidly expanding hematoma of the thyroid [6, 15]. The onset of symptoms of an expanding hematoma, such as a painful pre- and paratracheal swelling and dyspnoea, may be delayed in approximately 50% of the patients [7, 13], necessitating inhospital monitoring for more than 24 hours.

Thyroid function tests of the patient were indicative of primary hyperthyroidism. During the follow-up, her free T4 level recovered, while her TSH level remained slightly suppressed, which may be attributed to a delayed pituitary response, as evidenced from Jostel's TSH Index (7.16, 4.40, and 0.99 at days 1, 2, and 28, respectively). Given the lack of antibodies directed against the thyroid gland and the absence of evidence for a pre-existing thyroid disease, it is amenable that the hyperthyroxinemia in the patient could be explained by thyroid trauma. This case is special and interesting in such a way that it concerned a 17-year-old adolescent who did not experience symptoms suggestive of hyperthyroxinemia. In addition, the patient had no own or family history of thyroid disease. Despite the absence of hyperthyroid symptoms, thyroid function tests showed hyperthyroxinemia. The lack of hyperthyroid symptoms in our patient could be explained by the fact that she was young and previously healthy. For the publication of this case report, we obtained a written informed consent from our subject.

With this case report, we add that hyperthyroxinemia following a neck trauma may go unnoticed if hyperthyroid symptoms are mild or absent and thyroid function tests are not performed. Therefore, hyperthyroxinemia may occur more frequent after a blunt trauma of the neck than previously expected.

## Figures and Tables

**Table 1:** Thyroid function tests of the patient.

	Day 1	Day 2	Day 28	Reference range
TSH (mU/L)	0.46	0.14	0.33	0.5–4.3
FT4 (pmol/L)	59.0	47.3	15.6	12–22
Anti-TPO antibodies (kU/L)			<30	<60
Anti-TSH receptor antibodies (U/L)			<0.1	<1.8

TSH = thyroid stimulating hormone; FT4 = Free thyroxine; TPO = thyroid peroxidase.

## Data Availability

The data used to support the findings of this study are available upon request to either m.romijn1@amsterdamumc.nl or m.finken@amsterdamumc.nl.
